# *Lepidium sativum* Sprouts Grown under Elevated CO_2_ Hyperaccumulate Glucosinolates and Antioxidants and Exhibit Enhanced Biological and Reduced Antinutritional Properties

**DOI:** 10.3390/biom11081174

**Published:** 2021-08-09

**Authors:** Modhi O. Alotaibi, Galal Khamis, Hamada AbdElgawad, Afrah E. Mohammed, Mohamed S. Sheteiwy, Mudawi M. Elobeid, Ahmed M. Saleh

**Affiliations:** 1 Department of Biology, College of Science, Princess Nourah Bint Abdulrahman University, Riyadh 84428, Saudi Arabia; mouotaebe@pnu.edu.sa; 2 Department of Laser Applications in Metrology, Photochemistry and Agriculture (LAMPA), National Institute of Laser Enhanced Sciences, Cairo University, Giza 12613, Egypt; 3 Department of Botany and Microbiology, Faculty of Science, Beni-Suef University, Beni-Suef 62521, Egypt; hamada.abdelgawad@uantwerpen.be; 4 Department of Agronomy, Faculty of Agriculture, Mansoura University, Mansoura 35516, Egypt; salahco_2010@mans.edu.eg; 5 Department of Silviculture, Faculty of Forestry, University of Khartoum, Khartoum 13314, Sudan; emudawi2828@hotmail.com; 6 Department of Botany and Microbiology, Faculty of Science, Cairo University, Giza 12613, Egypt

**Keywords:** *Lepidium sativum*, elevated CO_2_, sprouting, antioxidant metabolites, glucosinolates, bioactivity

## Abstract

The nutritional and health-promoting properties of plants are largely determined by their tissue chemistry. Tuning growth conditions could affect the accumulation of phytochemicals and, therefore, enhance the biological activities. Herein, the impact of elevated CO_2_ (eCO_2_; 620 µmol CO_2_ mol^−1^ air) on growth and chemical composition of sprouts of three *Lepidium sativum* cultivars (Haraz, Khider and Rajab) was investigated. Changes in the sprout actions against some human chronic diseases were evaluated. eCO_2_ induced biomass accumulation (1.46-, 1.47- and 2-fold in Haraz, Khider and Rajab, respectively) and pigment accumulation and reduced the level of antinutrients in *L. sativum* cultivars. Compared to the control, eCO_2_ induced total glucosinolate accumulation (0.40-, 0.90- and 1.29-fold in Khider, Haraz and Rajab, respectively), possibly through increased amino acid production, and their hydrolysis by myrosinase. In line with increased polyphenol production, improved phenylalanine ammonia lyase activity was observed. The antioxidant, anti-inflammatory, hypocholesterolemic, antibacterial and anticancer activities of the produced sprouts were significantly improved by sprouting and eCO_2_ exposure. PCA indicated that the cultivars showed interspecific responses. Thus, the present study confirms the synergistic effect of sprouting with eCO_2_ exposure as a promising approach to produce more bioactive *L. sativum* sprouts.

## 1. Introduction

*Lepidium sativum* L. (garden cress), a member of Brassicaceae (Cruciferae), is an edible annual herb native to North Africa and West Asia, but currently it is cultivated globally [1,2]. *L. sativum* is one of the most common plant species of the genus *Lepidium*, due to its important uses in folk medicine [3]. Garden cress is available in markets as young live sprouts, being utilized as a valuable dietary supplement with a characteristic spicy flavor [4]. Sprouts are the young seedlings produced by germinating the seeds. They are collected and consumed before growing true leaves [5]. The sprouting process increases the levels of bioactive compounds and, thus, enhances the health-promoting properties [5,6]. The seeds of *L. sativum* comprise approximately 22.5% protein, 27.5% lipids, 30% dietary fiber and 1193 mg/100 g potassium, which constitutes the major mineral. Further, *L. sativum* seeds contain a considerable amount of essential amino acids and unsaturated fatty acids [1]. Unlike the seeds, the chemical composition of *L. sativum* sprouts is not fully characterized.

Various medicinal values were reported for *L. sativum,* such as antibacterial, anticancer, anti-inflammatory, analgesic, chemoprotective, hypocholesterolaemic, hypoglycemic, antidiabetic and antiasthmatic properties [1,2,4,7]. These biological activities were largely attributed to a variety of secondary metabolites such as isoflavonoids, phenolic acids and glucosinolates. Among these, glucosinolates are considered as the major class of secondary metabolites in *L. sativum* and other members of Brassicaceae [8]. Glucosinolates consist of a β-D-glucopyranosyl molecule bound to an N-hydroxyimino sulfate ester by a sulfur atom and modified by a side chain of amino acids [2]. In planta, glucosinolates play a direct role in defense against herbivores and pathogens, where upon tissue injury they are cleaved into glucose and isothiocyanates by the action of the myrosinase enzyme [9]. Interestingly, isothiocyanates have been reported to activate the human defense system against chronic diseases such as cancer and cardiovascular disorders [10]. In fact, isothiocyanates are more active than the parent glucosinolates that have no or little activity, therefore uncooked glucosinolate-rich plant foods, such as sprouts, are much more effective than cooked ones as they contain active myrosinase [11]. Further, isothiocyanates showed in vitro antibacterial, anticancer and anti-inflammation properties [12]. In addition to glucosinolates, the intake of plant foods rich in carotenoids, phenolic acids and flavonoids has been reported to reduce the risk of several human chronic diseases such as inflammation, diabetes, cancers and cardiovascular diseases [13]. In this connection, carotenoids such as carotene, lutein and xanthophylls are known as strong scavengers for reactive oxygen species (ROS) and to decrease inflammation [14]. The antimicrobial activities of carotenoids against *Escherichia coli*, *Micrococcus luteus*, *Bacillus subtilis* and *Vibrio parahaemolyticus* were reported [15]. Moreover, polyphenolic compounds were reported for their antioxidant, anticancer, anti-inflammatory, antimicrobial and hypocholesterolemic potentials [16,17].

Being the principal factor in determining the nutritional and health-promoting properties of plants, improving the accumulation of the bioactive metabolites in edible plants has been the scope of many studies [18]. Fortunately, tuning growth conditions has been proved as a powerful agricultural procedure to improve the growth and quality of herbal and medicinal plants [8]. In this aspect, recent publications have reported that growing plants under a CO_2_-enriched atmosphere could be beneficial in modulating tissue chemistry and, therefore, the nutritional and medicinal values of herbal and medicinal plants [5,6,19,20,21]. In fact, elevated CO_2_ (eCO_2_) could enhance the photosynthetic C assimilation, especially in C3 plant systems, and therefore supply the metabolic energy and precursors that are required to produce the bioactive metabolites. For instance, eCO_2_ enhanced the chemical composition and quality of peppermint (*Mentha piperita* L.), basil (*Ocimum basilicum* L.), dill (*Anethum graveolens* L.) and parsley (*Petroselinum crispum* L.), by altering carbon and nitrogen metabolism [20,21]. eCO_2_ enhanced the anti-inflammatory and anticancer properties of broccoli sprouts by enhancing photosynthesis and regulating sugar synthesis and breakdown [5]. Further, eCO_2_ improved the growth of alfalfa sprouts and increased vitamin, pigment, phenolic acid and flavonoid levels [6]. However, the improved plant yield in response to eCO_2_ could come at the cost of lower food quality as plants accumulate more carbohydrates and fewer minerals [22]. Although eCO_2_ could effectively enhance plant nutritive value, to the best of our knowledge, its effect on the chemical composition of *L. sativium* sprouts has not been previously studied.

Besides the lack of studies regarding the impact of eCO_2_ on *L. sativum* and the poor understanding of the tissue chemistry of its sprouts, the amazing health-promoting properties of *L. sativum* have motivated us to investigate the impact of eCO_2_ on the growth and the nutritional and medicinal values of *L. sativum* sprouts. Here, we are aiming to give in-depth details on how eCO_2_ improves the tissue composition of *L. sativum* sprouts. Metabolic profiling of antioxidant metabolites (carotenoids, phenolic acids and flavonoids) and glucosinolates and quantification of antinutrient (tannins, phytate, cyanide and oxalate) were performed. The activities of the key enzymes in phenolic biosynthesis, phenylalanine ammonia lyase (PAL), and glucosinolate hydrolysis, myrosinase, were assessed. Further, the provoked changes in the antioxidant (FRAP, ABTS and DPPH free radical scavenging), anti-inflammatory (inhibition of lipoxygenase and cyclooxygenase-2), hypocholesterolemic (micellar solubility inhibition and lipase and amylase inhibition), anticancer and antibacterial activities of the produced sprouts were assessed.

## 2. Material and Methods

### 2.1. Plant Material and Experimental Conditions

Seeds were collected from three *L. sativum* varieties (Haraz, Rajab and Khider). Voucher specimens were deposited in the herbarium of Beni-Suef University, Egypt at no. 154–156 and rinsed thrice with distilled water. Thereafter, the seeds were immersed for 1 h in sodium hypochlorite (5 g L^−1^) for surface sterilization of *L. sativum* seeds. Subsequently, the seeds were soaked for 2 h in distilled water then the water was removed. Next, the seeds were evenly dispersed on trays covered with vermiculite and moistened with 150 mL of aquaponic water. The trays were incubated in a growth chamber in controlled conditions (25 °C air temperature, 16 h light/8 h dark cycle, 400 µmol m^−2^ s^−1^ photosynthetically active radiation and relative humidity of 60%). The current ambient CO_2_ concentration (415 μmol CO_2_ mol^−1^ air) and the future climate CO_2_ (625 μmol CO_2_ mol^−1^ air), according to the predicted IPCC-SRES B2 scenario of eCO_2_ levels in 2100 [23], were selected. The different climate conditions were as follows: (1) ambient CO_2_ (aCO_2_, 415± 34 μmol CO_2_ mol^−1^ air); (2) eCO_2_ (625 ± 53 µmol CO_2_ mol^−1^ air). A CO_2_ analyzer was employed for observing the CO_2_ concentration. Thirty trays were used for the two treatments (15 trays/treatment), with 10 seeds in each tray. After 10 days, sprouts were collected from each tray and the fresh mass was measured. Liquid nitrogen was used for freezing sprouts and the samples were kept at −80 °C until biochemical assessment. Five biological replicates were carried out for each measurement, and 10 plants of the same tray were pooled for each biological replicate.

### 2.2. Evaluation of the Contents of Pigments

Separation and quantification of pigment contents in the sprouts were performed according to Al Jaouni et al. [21]. The frozen sprouts were extracted in acetone with the aid of a MagNALyser (Roche, Vilvoorde, Belgium). Thereafter, centrifugation at 14,000× *g* for 20 min at 4 °C was performed for the mixture and supernatant was subsequently filtered (Acrodisc GHP filter, 0.45 μm 13 mm). For analyzing the concentrations, HPLC (Shimadzu SIL10-ADvp, reversed phase) was used. A diode array detector (Shimadzu SPD- M10Avp) was used for chlorophyll, carotene and xanthophyll evaluation at 420, 440, 462 and 660 nm.

### 2.3. Amino Acid Determination

Amino acids were determined from 200 mg of sprouts combined with 1 mL of 80% (*v*/*v*) aqueous ethanol [24]. For accurate evaluation and correction of varied mass spectrometry reactions, norvaline was employed as an internal standard. The homogenate was centrifuged for 30 min at 14,000× *g*. The supernatant was added to fresh tubes and then dried. Next, chloroform (1 mL) was used for pellet resuspension followed by centrifugation (14,000× *g* for 30 min), then further extraction of the plant in water was performed and the extract was blended with the chloroform-suspended pellet. Then, the combined extract was centrifuged at 20,000× *g* for 10 min. Thereafter, the aqueous phase was filtered (Millipore microfilters, 0.2 μm pore size) for amino acid assessment. Amino acids were separated on a BEH amide 2.1 × 50 column (Waters Acquity UPLC-tqd system, Milford, MA, USA) [21]. A 10 μL sample was inserted and elution was carried out with a gradient of 0.1% formic acid (FA) in H_2_O and 0.1% formic acid in acetonitrile. The sample was kept at 20 °C and at a 30 °C column temperature.

### 2.4. Evaluation of Phenol Content

Phenolic compounds were quantified by UHPLC-MS/MS analysis (Saleh et al., 2018). Dried powdered tissues were extracted in 80% (*v*/*v*) ethanol at 70 °C for 30 min. Subsequently, the extract was centrifugated at 12,000× *g* for 30 min, and the clear supernatant was collected and concentrated by using a rotary evaporator. Then, the precipitate was added to methanol (HPLC grade) to a final concentration of 1000 ppm. Furthermore, for chromatographic analysis of the extract, the Acquity UPLC System (Waters, Milford, CT, USA) was employed. For the separation, the Acquity BEH C18 column (100 mm × 2.1 mm) was used. The mobile phase elements were eluent A: ultrapure water comprising formic acid (0.1%) and eluent B: acetonitrile. The flow rate was 0.2 mL/min, and 20 μL samples were injected. A linear gradient of eluents, a gradual increase in ratio of eluent B from 3 to 100% in 10 min, was applied. 3,5-dichloro-4- hydroxybenzoic acid was employed as the internal standard.

### 2.5. Glucosinolate Extraction and Determination

Extraction of glucosinolates was carried out according to [5]. For inhibition of myrosinase enzyme activity, samples were steamed for two min prior to glucosinolate extraction. One gram of plant sample was homogenized with the aid of a porcelain mortar (Sigma, Antwerp, Belgium) in 3 mL of 70% (*v*/*v*) aqueous MeOH supplemented with trifluoroacetic acid (TFA, 1.5 g/L). The extracts were then transported to stoppered Erlenmeyer flasks at 70 °C for 30 min to be conditioned with continuous agitation in a thermostatic bath. The extracts were cooled and centrifugated for 20 min at 8000× *g*. The supernatants were filtered and then evaporated to dryness at 40 °C, thereafter the dry precipitate was resuspended in 0.2 mM HEPES–KOH (pH 7.0). To evaluate the enzymatic activities, 10 μL of total glucosinolate extract was mixed with thioglucosidase (0.12 U) at 37 °C for 24 h. Perchloric acid solution (18 mM) was added to terminate the reaction. For measuring the control samples, extracts were substituted by buffer. Glucose obtained through thioglucosidase from glucosinolate hydrolysis was assessed stoichiometrically, where 1 mol of total glucosinolates is equal to 1 mol of produced glucose. Total glucose was determined by a glucose oxidase/peroxidase kit (GAGO20-1KT, Sigma) and allyl-glucosinolate and sinigrin were employed as a positive control and as a calibrant, respectively.

### 2.6. Evaluation of Myrosinase Enzyme Activity

Samples of *L. sativum* sprouts of each variety under eCO_2_ or aCO_2_ (control) treatment were added to 0.1 M phosphate buffer (pH 6.0) containing benzamidine (5 mM). The mixture was kept at 4 °C for 30 min with gentle continuous stirring. The centrifuged (15 min, 13,000× *g*) supernatant was collected, filtered and precipitated in 70% saturated ammonium sulfate. Next, 10 mM phosphate buffer was added to dissolve the pellet and dialyzed against 10 mM phosphate buffer, pH 7.0. The dialyzate was applied to a DEAE-Sephadex column equilibrated with phosphate buffer and the elution of the protein was carried out with the same phosphate buffer supplemented with 0.1 M NaCl. Following the method adopted by Schwimmer and Weston [25], myrosinase activity was determined by evaluating the hydrolysis of sinigrin (Sigma) by observing absorbance reduction (227 nm).

### 2.7. Inhibition of Micellar Solubility of Cholesterol

The impact of *L. sativum* sprout extract on cholesterol micellar solubility was assessed as described previously [19]. Concentrated extracts from sprouts, treated and untreated with eCO_2_, were added to 7 mL of micellar solution containing cholesterol (2 mM), sodium taurocholate (10 mM), NaCl (132 mM), oleic acid (5 mM) and sodium phosphate (15 mM, pH 7.4) at the rate of 10 mg/mL. Sonication for 2 min was performed on the mixture, followed by incubation for 24 h in a water bath at 37 °C. Afterwards, ultracentrifugation for 60 min at 20 °C and 40,000× *g* was carried out. Then, 10 µL of the collected supernatant was utilized for spectrophotometric determination (500 nm) of cholesterol concentration using a cholesterol determination kit (Pointe Scientific, C7510). Inhibition of micellar solubility was calculated using the following equation: Inhibition activity (%) = [(C−S)/C] × 100, where C = cholesterol concentration in control micellar solution and S = cholesterol concentration in micellar mixture mixed with the test samples.

### 2.8. Lipase and Amylase Inhibition Assays

The efficacy of sprout extracts as inhibitors for pancreatic lipase was assessed by using 4-Methylumbelliferyl oleate (4-MUO) as a substrate in accordance with [19]. Different concentrations of seed extract (0.5 mL) were added to 0.5 mL of fresh lipase, lipase from porcine pancreas (1 mg/mL, Sigma). The mixture was stirred for 10 min followed by centrifugation for 10 min at 4000× *g*, then 2 mL 4-MUO (0.1 mM) solution was added. The reaction medium without extract was applied as a blank. About 0.2 mL were then taken at various time points, and 4-MUO hydrolysis by lipase was evaluated at wavelengths of 350 nm and 450 nm. To compute IC_50_ values (extract concentration that suppresses 50% the pancreatic lipase activity), a logarithmic regression curve was made.

Furthermore, the inhibitory effect of α-amylase was assessed for each extract by applying starch as a substrate. In brief, the reaction medium was composed of 1 mL of extract solution at various levels and 1 mL of α-amylase (10 mg/mL) in buffer. This combination was kept for 10 min in a 37 °C incubator. Following pre-incubation, the reaction was started when 1 mL of potato starch solution (0.5% in water boiled for 15 min at 100 °C) was added. Incubation was completed by the addition of 0.1 mL of 50 mM HgCl_2_ after 30 min. The generated reducing sugars were determined calorimetrically by Nelson’s method. To avoid potential errors caused by the presence of endogenous reducing sugars in the extract, a negative control was performed with boiled enzyme.

### 2.9. Antioxidant Activity

Antioxidant potential was evaluated by ferric reducing antioxidant power (FRAP), 2,2′-Azino-bis(3-ethylbenzothiazoline-6-sulfonic acid) (ABTS) and 2,2-diphenyl-1-picryl-hydrazyl-hydrate (DPPH) according to [21,26]. Powdered *L. sativum* sprouts were extracted by aqueous ethanol (80%, *v*/*v*) and centrifuged for 20 min at 14,000× *g*. Subsequently, antioxidant activity assessment was performed by combining 0.1 mL diluted sprout extracts with 0.25 mL of the DPPH, ABTS or FRAP reagents. Following incubation at 25 °C, absorbance was spectrophotometrically measured (517 nm and 600 nm).

### 2.10. Lipoxygenase (LOX) Assay

Antilipoxygenase activity was determined via using the LOX enzyme and linoleic acid as a substrate. LOX (400 U/mL) was mixed with 10 µL from each *L. sativum* sprout extract and maintained in the dark for 5 min at 25 °C. A volume of linoleic acid solution (0.4 mM) was added to initiate the reaction (20 min at 25 °C). Then, 100 µL of fresh ferrous orange xylenol (FOX) reagent was added. The reaction was carried out at 25 °C for 30 min and the absorbance was assessed at 560 nm. The inhibition percentage was calculated.

### 2.11. Evaluation of Cyclooxygenase 2

A cyclooxygenase 2 (COX) assay was performed using a COX assay kit (Cayman Chemical Company, Ann Arbor, MI, USA). A microtiter plate was covered and shaken (18 h at room temperature). The plate was maintained in dark conditions for 1.5 h at 25 °C. Afterwards, the reading was obtained at 420 nm and the inhibition percentage was estimated.

### 2.12. Antibacterial Activities

The sprout ethanolic extracts of each cultivar were tested for their antibacterial effect against *Streptococcus sp*, *E. coli*, *B. subtilis*, *P. aeruginosa* and *S. lutea* using the disc diffusion method. A suspension (10^6^ CFU/mL) of each test bacterium was distributed on Mueller–Hinton agar. The sterilized filter paper discs were loaded with extracts (5 μg/disc). Ethanol was applied as a negative control. Prepared discs were put on agar plates at 37 °C for 24 h. A Vernier caliper was used to measure the inhibition zones.

### 2.13. Anticancer Activities

The cytotoxic effect of *L. sativum* sprout extracts (2 mg/mL) was investigated against four human cancer cell lines, colon carcinoma, hepatocellular carcinoma, urinary bladder carcinoma and embryonic kidney adenocarcinoma. Cell viability was assessed by using CellTiter-Blue reagent (Promega, Madison, WI, USA) and the results were presented as % dead cells [27].

### 2.14. Statistical Analyses

Data analyses were performed using the procedures provided in Statistical Analysis System (PASW statistics version 18.0, SPSS Inc., Chicago, IL, USA). Data normality was checked using the Kolmogorov–Smirnov test and the homogeneity of variances was tested by Levene’s test. A Student’s *t*-test at a probability level of 0.05 was used to test the difference between means of the same cultivar in the two CO_2_ scenarios. Tukey’s test (*p* < 0.05) was carried out for separation of means of the different cultivars within the same CO_2_ level. Further, to test the interaction between CO_2_ levels and cultivars in the measured parameters, a two-way ANOVA was performed (Supplementary Table S1). The number of replicates for each experiment was five (*n* = 5). OriginLab software (OriginLab 9, Northampton, MA, USA) was used for performing principal component analysis (PCA) of the full dataset.

## 3. Results and Discussion

### 3.1. Elevated CO_2_ Improves the Biomass Production of L. sativum Sprouts

**Figure 1:**
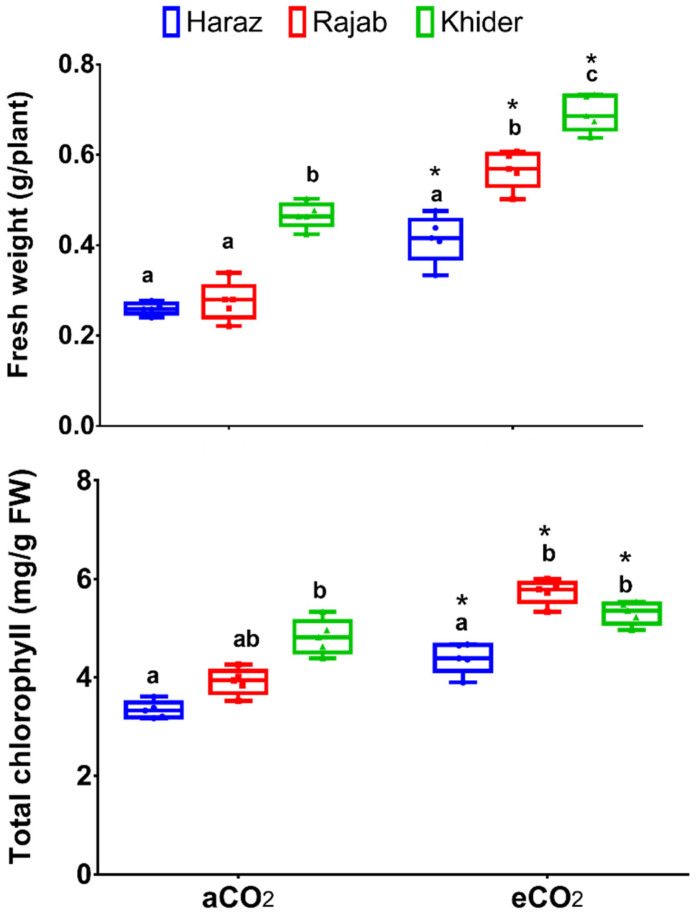
Fresh biomass and total chlorophyll content in sprouts of three *Lepidium sativum* cultivars grown under either aCO_2_ (410 μmol CO_2_ mol^−1^ air; control) or eCO_2_ (620 µmol CO_2_ mol^−1^ air). Values are presented as means ± standard error of 5 independent replicates. Different letters above the boxes at the same CO_2_ level indicate significant differences at the 0.05 probability level as revealed by Tukey’s test. * Indicates significant changes (*p* < 0.05) compared to the corresponding control, as revealed by Student’s *t*-test.

Elevated CO_2_ has been used as a growth promoter for a variety of herbal and crop plants [19,20,21]. Herein, the bio-fertilizing effect of eCO_2_ (627 µmole CO_2_ mol^−1^ air) on the growth, the levels of bioactive metabolites and the bioactivity of *L. sativum* cultivars was assessed and compared to the control conditions (aCO_2_, 410 μmol CO_2_ mol^−1^ air). Regarding the growth of *L. sativum* sprouts, eCO_2_ significantly (*p* < 0.05) improved the fresh mass of the three cultivars (Haraz, Khider and Rajab) by about 1.46-, 1.47- and 2-fold, respectively, relative to the corresponding controls (Figure 1). Further, eCO_2_ significantly increased the total chlorophyll content in the three *L. sativum* cultivars. Similarly, CO_2_ enrichment was reported to significantly increase the growth parameters of broccoli [5], alfalfa [6], dill and parsley [20]. In fact, eCO_2_, being a substrate for Calvin–Benson cycle, could improve C fixation by enhancing the carboxylation reaction at the expense of the oxygenation reaction of RuBisCO (ribulose-1,5-bisphosphate carboxylase/oxygenase) [21]. In agreement, the enhanced biomass, by eCO_2_ treatment, was accompanied by significant increases in chlorophyll content and net photosynthesis rate in basil, peppermint, wheat, maize and alfalfa [6,21,28,29].

### 3.2. L. sativum Sprouts Produced under eCO_2_ Showed Reduced Content of Antinutrients

Previous reports have showed that subsequent free-air CO_2_ enrichment had decreased antinutrient concentrations in several plant species such as soybean, sorghum and wheat [30,31]. The present results showed that eCO_2_ treatment significantly decreased phytate in the three *L. sativum* cultivars, tannins in Haraz and Rajab, cyanide in Rajab and Khider and oxalate in Rajab only, compared to the aCO_2_ condition (Table 1). As antinutritional factors induce great effects on mineral bioavailability in foods [32], the observed decreases in phytate and oxalate indicate the high nutritive value of eCO_2_-treated *L. sativum*. For instance, phytate, a phosphate storage molecule, inhibits the absorption of dietary minerals such as zinc in the human gut [33]. In contrast, other studies have shown that eCO_2_ enhanced or did not affect the oxalate level in ponderosa pine [34] and lupin [35], respectively.

### 3.3. The Levels of Amino Acid Precursors for Glucosinolates and Phenolics Are Enhanced by eCO_2_

**Figure 2:**
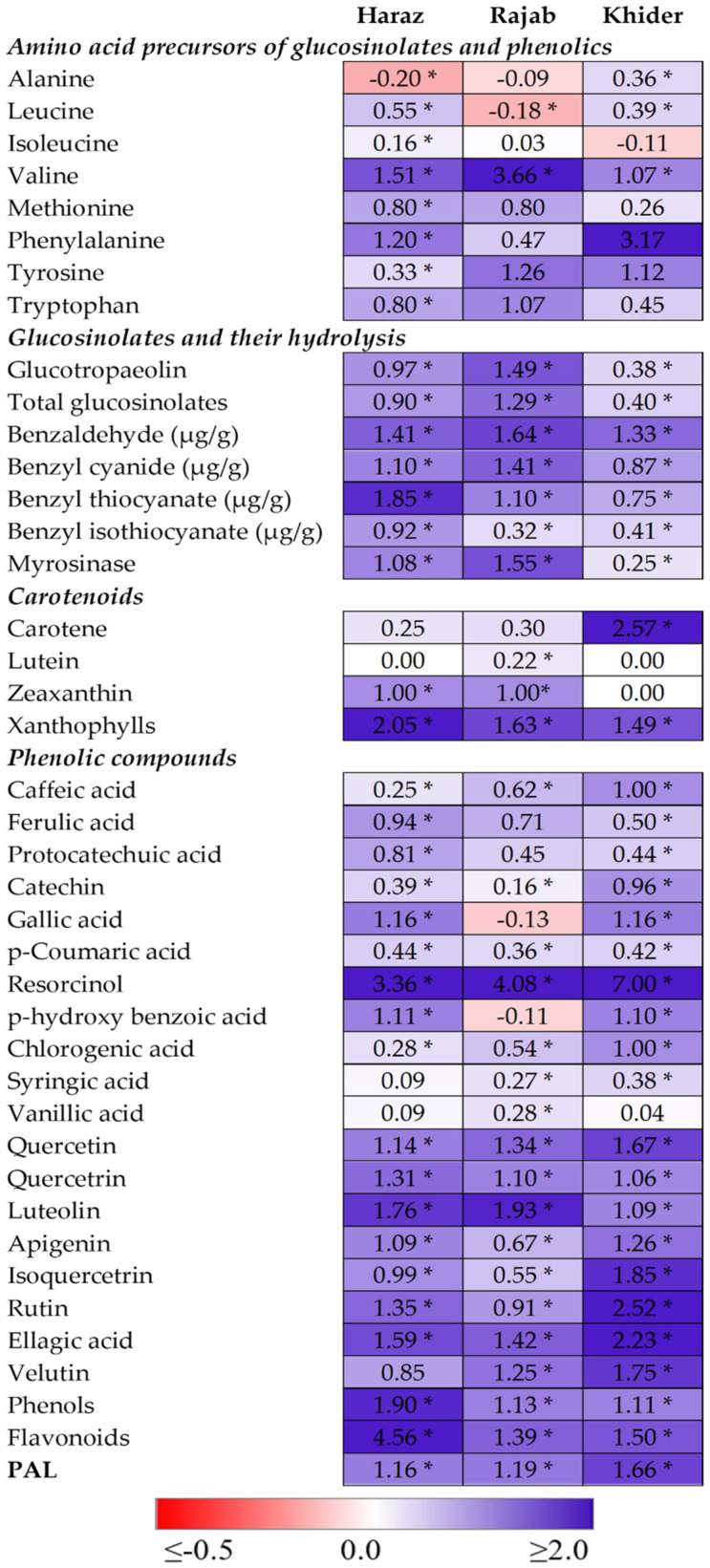
Heatmap of fold change ratios in antioxidant metabolites (carotenoids, phenolic acids and flavonoids) and glucosinolates and the activities of PAL and myrosinase in sprouts of three *Lepidium sativum* cultivars in response to eCO_2_ (620 µmol CO_2_ mol^−1^ air). Fold change in each metabolite was calculated relative to its corresponding mean (*n* = 5) in the aCO_2_-grown (410 μmol CO_2_ mol^−1^ air) plant. As shown in the color scale, blue indicates improvement, white no change and red inhibition. * Indicates significant changes (*p* < 0.05) compared to the corresponding control, as revealed by Student’s *t*-test.

It is well known that the content of essential amino acids is one of the most important factors that determine the nutritional value of plants [20,21]. For instance, methionine is a key factor in human nutrition due to its role in choline extraction, lipid metabolism and activation of the antioxidant defense system [36]. Further, free amino acids are the precursors for the biosynthesis of several health-promoting substances, such as glucosinolates and phenolics. Phenylalanine, tyrosine and tryptophan are the precursors for indolic and aromatic glucosinolates, while alanine, leucine, isoleucine, valine and methionine are the precursor for aliphatic glucosinolates [37]. Further, phenylalanine is the substrate for the key enzyme in phenolic biosynthesis, phenylalanine ammonia lyase (PAL), via the phenylpropanoid pathway [38]. Therefore, the synthesis of the bioactive metabolites is closely related to the availability of their amino acid precursors. Herein, eCO_2_ treatment significantly increased (*p* < 0.05) the levels of valine, methionine, phenylalanine, tyrosine and tryptophane in the three cultivars, compared to the corresponding control values (Figure 2, Supplementary Table S2). Further, eCO_2_ improved the levels of leucine in Haraz and Khider and that of alanine in Khider only. This positive impact of eCO_2_ on amino acid biosynthesis could be attributed to the upregulation of photosynthesis and, therefore, the increased availability of non-structural carbohydrates that provide C skeletons and metabolic energy required for the biosynthesis of amino acids [39]. In line with our findings, eCO_2_ treatment significantly improved the concentrations of essential and non-essential amino acids in peppermint, basil, dill and parsley [20,21].

### 3.4. The Accumulation and Hydrolysis of Glucosinolates Are Induced by eCO_2_

Glucosinolates are considered as the major bioactive constituent in *L. sativum* (Oszmiański et al., 2013). Our data showed that *L. sativum* sprouts contained considerable levels of glucotropaeolin, benzaldehyde and total glucosinolates that were enhanced significantly (*p* < 0.05) in the three cultivars in response to eCO_2_ treatment (Figure 2, Supplementary Table S2). For instance, the total glucosinolates were boosted by 2.3-, 1.9- and 1.4-fold in Rajab, Haraz and Khider cultivars when affected by eCO_2_ treatments, respectively, compared to the control. Such enhancement in glucosinolates matches with the increased content of their amino acid precursors in *L. sativum* sprouts (Figure 2, Supplementary Table S2). Coelho et al. (2007), reported that *L. sativum* contained one principal glucosinolate, glucotropaeolin, which is an aromatic glucosinolate and its sidechain is synthesized from phenylalanine. Upon tissue injury, the compartmentalization of the myrosinase enzyme and its substrate is broken to produce benzyl-isothiocyanates that are much more reactive than the parent glucosinolates [11]. Interestingly, eCO_2_ significantly promoted the activity of myrosinase in the three cultivars, which was consistent with the increased isothiocyanate levels. In this context, eCO_2_ treatment increased the levels of the predominant glucosinolate, glucoraphanin, and total glucosinolates and enhanced the activity of myrosinase in broccoli sprouts [5]. eCO_2_ enhanced the levels of aliphatic glucosinolates in Chinese kale under N supply up to 10 mmol L^−1^ N, but a negative impact was observed at higher N levels [40].

### 3.5. Elevated CO_2_ Induces the Accumulation of Antioxidant Metabolites

Being a major cause of chronic diseases, keeping the levels of reactive oxygen species (ROS) under control is of immense importance for human health [41]. In this regard, several classes of phytochemicals, such as carotenoids, phenolic acids and flavonoids, were reported to have meaningful antioxidant capacities [13,14]. In fact, the synthesis of the various classes of secondary metabolites in plants is in close connection with C and N metabolism [42]. Therefore, by its positive impact on C and N metabolism, eCO_2_ could enhance the accumulation of these phytochemicals [20,21]. In the current study, the impact of eCO_2_ on the accumulation of carotenoids, phenolic acids and flavonoids in the sprouts of three *L. sativum* cultivars was evaluated (Figure 2, Supplementary Table S2). Regarding carotenoids, the exposure of *L. sativum* sprouts to eCO_2_ increased the levels of carotene and xanthophylls in the three cultivars, whereas the highest fold change for carotene was observed in Khider (2.57-fold) and that of xanthophylls was recorded in Haraz (2.05-fold). On the other hand, the level of lutein increased in Rajab only and zeaxanthin improved in Haraz and Rajab. Similarly, Saleh et al. [28] reported that eCO_2_ (620 ppm) significantly increased the total carotenoid level in maize plants. However, the same concentration of CO_2_ did not cause any significant change in carotenoid levels in wheat [43]. Further, Dhami et al. [44] reported that the response of carotenoids to eCO_2_ in Arabidopsis leaves is age dependent, where the younger leaves were more responsive and accumulated more than double the carotenoids of older ones. Thus, the impact of eCO_2_ on carotenoid biosynthesis is somewhat variable depending on the species and age of the tested plants.

Among the detected 19 phenolic compounds in *L. sativum* sprouts, p-hydroxy benzoic acid was the dominant phenolic acid, while quercetin and rutin were the predominant flavonoids (Figure 2, Supplementary Table S2). eCO_2_ treatment significantly improved the levels of the majority of the detected phenolics in the three cultivars, except for gallic and p-hydroxy benzoic acid in Rajab, vanillic acid in Haraz and Khider and syringic acid in all cultivars. The most induced phenolic compound by eCO_2_ was resorcinol, as it was increased by 3.36-, 4.08- and 7.00-fold in Haraz, Rajab and Khider cultivars, respectively. Further, the total flavonoids and polyphenolic contents were significantly increased in the three cultivars affected by eCO_2_, compared to the corresponding controls. The improvements in flavonoid content were more pronounced than those of total polyphenols in all cultivars. For instance, the eCO_2_-induced accumulation of flavonoids and total polyphenols in the Haraz cultivar were 4.56- and 1.90-fold, respectively, relative to the corresponding control values (Figure 2, Supplementary Table S2). It is worth mentioning that the increments in the content of polyphenolic compounds in the three *L. sativum* sprouts were consistent with the enhanced levels of phenylalanine, the substrate for PAL. Similarly, the study by Hartley et al. [45] found a positive correlation between polyphenolic compounds and PAL content under elevated eCO_2_. Accordingly, the positive impact of eCO_2_ on the biosynthesis and accumulation of phenolics has been reported in several medicinal and crop plants, such as dill and parsley [20], basil and peppermint [21], fenugreek [19] and alfalfa [6].

### 3.6. The eCO_2_-Induced Accumulation of Antioxidants and Glucosinolates Is Accompanied by Promoted Bioactivity

**Figure 3:**
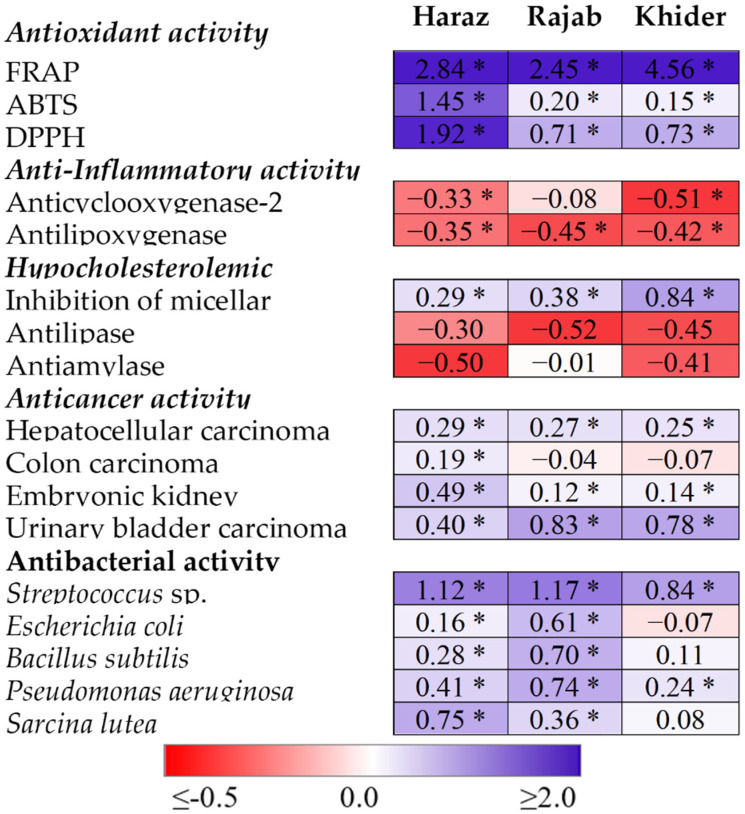
Heatmap of fold change ratios in antioxidant, anti-inflammatory, hypocholesterolemic, anticancer and antibacterial activities of sprouts of three *Lepidium sativum* cultivars in response to eCO_2_ (620 µmol CO_2_ mol^−1^ air). Fold change in each activity was calculated relative to its corresponding mean (*n* = 5) in the aCO_2_-grown (410 μmol CO_2_ mol^−1^ air) plants. As shown in the color scale, blue indicates improvement, white no change and red inhibition. * Indicates significant changes (*p* < 0.05) compared to the corresponding control, as revealed by Student’s *t*-test.

The biological activities of medicinal plants are largely correlated with their endogenous bioactive phytochemicals. Therefore, by its positive impact on accumulation of glucosinolates and antioxidant metabolites, eCO_2_ is expected to affect the bioactivity of *L. sativum* sprouts. Herein, the antioxidant, anti-inflammatory (inhibition of lipoxygenase and cyclooxygenase-2), hypocholesterolemic (inhibition of micellar solubility), anticancer and antibacterial activities of *L. sativum* sprouts grown under aCO_2_ and eCO_2_ were assessed (Figure 3, Supplementary Table S3). eCO_2_ significantly improved the antioxidant capacities of the three *L. sativum* cultivars, where the highest fold change (4.56) in FRAP radical-scavenging activity was observed in Khider and those of ABTS and DPPH radicals (1.45 and 1.92, respectively) were obtained in the Haraz cultivar. The IC_50_ values of lipoxygenase inhibition were significantly decreased in all *L. sativum* cultivars, however, that of cyclooxygenase-2 was significantly decreased Haraz and Khider only, relative to their values in the corresponding control. For the three cultivars, *L. sativum* sprouts produced under eCO_2_ showed significantly higher activity against three human cancer cell lines (hepatocellular, embryonic kidney and urinary bladder). However, eCO_2_ did not affect the activity of *L. sativum* sprouts against colon carcinoma, except for Haraz. Further, eCO_2_ treatment significantly improved the activity of *L. sativum* sprouts against the tested bacterial species with three exceptions, where no significant changes were observed in the case of *Bacillus subtilis* in Rajab and Khider and for *Sarcina lutea* in Khider. In line with our study, previous studies have reported the positive impact of eCO_2_ on the anti-inflammatory, antioxidant, hypocholesterolemic, anticancer and antibacterial activities of several herbal and medicinal plants [6,19,20,21]. The authors attributed the enhanced biological activities, as affected by eCO_2_, to the improved levels of the bioactive metabolites, carotenoids, vitamins, polyphenols and/or glucosinolates. Moreover, numerous studies have documented the significant antioxidant, anti-inflammatory, hypolipidemic, anticancer and antibacterial activities of *L. sativum* [1,2,4,7]. The results in Figure 3 and Supplementary Table S3 show that the lipase activity was significantly inhibited in all studied cultivars of *L. sativum* when treated with eCO_2_ as compared with aCO_2_ treatment. Similarly, the antiamylase property was also significantly improved in all studied cultivars upon treatment with eCO_2_, except in the Rajab cultivar (Figure 3, Supplementary Table S3). α-glucosidase, lipase and α-amylase are known as the key enzymes for the hydrolysis of fat and carbohydrates, respectively [46]. Thus, lipase- and amylase-induced inhibition of triglyceride and carbohydrate absorption can be an approach for the prevention of obesity [46].

### 3.7. L. sativum Cultivar-Specific Response to eCO_2_

**Figure 4:**
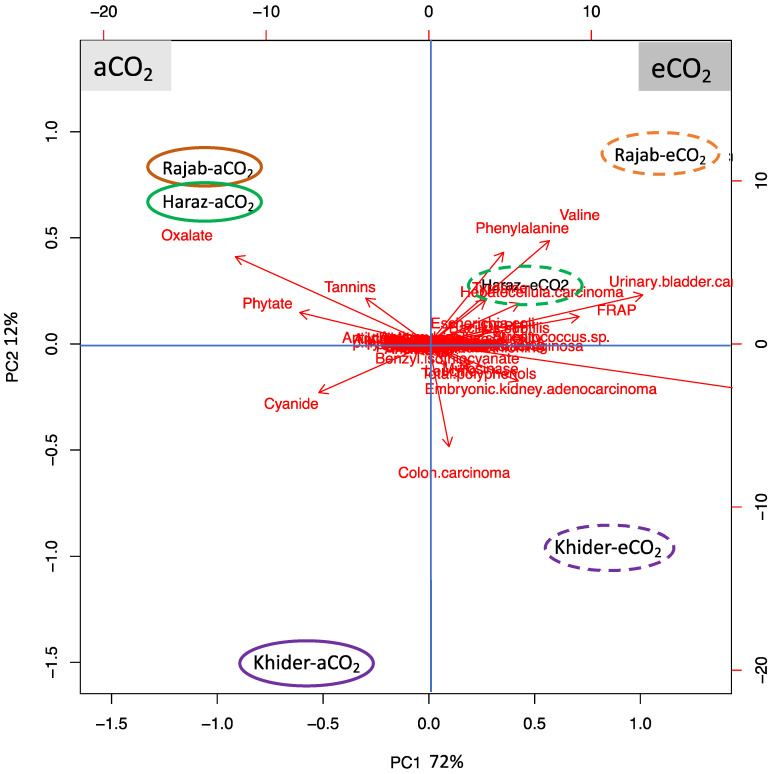
Principal component analysis of the average levels of assayed metabolite and biological activities in sprouts of three *Lepidium sativum* cultivars grown under either aCO_2_ (410 μmol CO_2_ mol^−1^ air; control) or eCO_2_ (620 µmol CO_2_ mol^−1^ air). Variances explained by the first two components (PC1 and PC2) appear in parentheses. The PCA is based on Z-score normalized data. Green, purple and orange circles represent Rajab, Haraz and Khider samples, respectively. Clusters with a dashed or solid outline represent samples grown under eCO_2_ or aCO_2_, respectively. The circles were arbitrary.

To test the specific responses of different *L. sativum* cultivars to eCO_2_, we preformed principal component analysis (PCA) (Figure 4). The biplot shows uniform parameters along the first two dimensions that represent about 72% and 12% of the data variability, respectively (Figure 4). PC1 clearly separates the measured parameters on the basis of eCO_2_ treatment (72% of the data variables), while the cultivar-specific responses were resolved along PC2 (12% of data variables). PC2 separated the changes in the Khider cultivar from the changes in Rajab and Haraz cultivars, however this separation was lacking between Rajab and Haraz cultivars. PCA indicates that eCO_2_ particularly increased valine and phenylalanine, reduced cyanide in treated Rajab, improved anticancer properties, e.g., in colon carcinoma, and reduced antinutrients oxalate, phytate and tannins in treated Khider.

## 4. Conclusions

Based on the findings presented in this research, it could be concluded that growing *L. sativum* sprouts under eCO_2_ (620 µmol CO_2_ mol^−1^ air) is a powerful approach to improve its chemical composition. The eCO_2_-induced accumulation of glucosinolates and polyphenols could be ascribed to the enhanced availability of their amino acid precursors and the upregulation of the key enzymes in their biosynthesis, myrosinase and PAL, respectively. The enhanced accumulation of bioactive compounds was accompanied by improved health benefits. Although the three *L. sativum* cultivars used in this study responded positively to eCO_2_, cultivar-specific responses and quantitative variations in the levels of many of the assessed compounds were observed. These results increase the potentiality of using *L. sativum* sprouts grown under high CO_2_ as a promising nutritional and health-promoting functional food or food additive. Further, more molecular studies to uncover the mechanisms that underlie the modulation of secondary metabolites, particularly glucosinolates, are needed in future to validate the potentiality of eCO_2_ in improving sprouting processes of economically important plant seeds.

## Figures and Tables

**Table 1:** Levels of antinutrients in sprouts of three *Lepidium sativum* cultivars grown under either aCO_2_ (410 μmol CO_2_ mol^−1^ air; control) or eCO_2_ (620 µmol CO_2_ mol^−1^ air).

Antinutrients	aCO_2_			eCO_2_		
	Haraz	Rajab	Khider	Haraz	Rajab	Khider
Tannins	32.1 ± 1.1 c	27.3 ± 1.2 b	19.3 ± 2.4 a	22.6 ± 2.4 B *	18.2 ± 2.1 AB *	15.4 ± 5.1 A
Phytate	87.4 ± 1.1 a	91.4 ± 4.7 a	86.7 ± 5.2 a	77.1 ± 4.0 A *	72.1 ± 9.2 A *	61.8 ± 9.0 A *
Cyanide	69.1 ± 2.7 a	70.8 ± 4.1 a	85.4 ± 2.5 b	72.1 ± 4.2 B	55.1 ± 2.2 A *	51.8 ± 1.9 A *
Oxalate	112.7 ± 11.6 b	123.7 ± 8.7 b	91.5 ± 5.1 a	101.2 ± 8.2 B	77.5 ± 4.7 A *	84.1 ± 5.1 A

Values are presented as means ± standard error of 5 independent replicates. Different letters in the same row and the same CO_2_ level indicate significant differences at the 0.05 probability level as revealed by Tukey’s test. * Indicates significant changes (*p* < 0.05) compared to the corresponding control, as revealed by Student’s *t*-test.

## Data Availability

Data presented in this study are available on reasonable request.

## Supplementary Materials

The following are available online at https://www.mdpi.com/article/10.3390/biom11081174/s1, Table S1: A two-way ANOVA for the effect of CO2 levels, cultivars of and their interaction on the assessed parameters in L. sativum sprouts, Table S2: Levels of antioxidant metabolites, glucosinolates, their amino acid precursors and activities of phenylalanine ammonia lyase and myrosinase in the sprouts of three L. sativum cultivars grown under either aCO2 or eCO2, Table S3: Antioxidant, anti-inflammatory, hypocholesterolemic, anti-cancer and antibacterial activities of the sprouts of three Lepidium sativum cultivars grown under either aCO2 or eCO2.
