# Supplementary material for: Lepidium sativum Sprouts Grown under Elevated CO2 Hyperaccumulate Glucosinolates and Antioxidants and Exhibit Enhanced Biological and Reduced Antinutritional Properties
Source: Biomolecules. 2021 Aug 9;11(8):1174. doi: 10.3390/biom11081174 (PMC8392048; doi:10.3390/biom11081174)
Supplement: Supplementary file 1 [file biomolecules-11-01174-s001.zip › biomolecules-1294549-supplementary.pdf]

Supplementary Table S1: A two-way ANOVA for the effect of CO2 levels, cultivars of and their interaction on the assessed parameters in *Lepidium sativum* sprouts (numbers represent F values; ns, non-significant; *, P < 0.05; **, P < 0.01; ***, P < 0.001).

Parameters	Treatment (T)	Cultivar (C)	T X C
Fresh weight	58.215***	19.848***	3.156ns
Total Chlorophyll	69.492***	8.86**	16.849***
Alanine	0.159ns	7.74**	1.16ns
Leucine	2.794ns	1.116ns	2.463ns
Isoleucine	0.22ns	3.939*	1.345ns
Valine	46.976***	22.305***	11.863**
Methionine	67.917***	0.31ns	3.96*
Phenylalanine	48.805***	33.065***	5.52*
Tyrosine	4.98*	1.079ns	0.401ns
Tryptophan	181.046***	47.624***	12.326**
Glucotropaeolin	89.678***	18.878***	2.199ns
Total glucosinolates	126.742***	5.747*	5.918*
Benzaldehyde	22.962***	0.725ns	0.27ns
Benzyl cyanide	110.348***	20.485***	0.395ns
Benzyl thiocyanate	132.001***	2.156ns	5.108*
Benzyl isothiocyanate	101.374***	34.907***	7.973**
Myrosinase	202.48***	103.932***	23.185***
Xanthophylls	131.716***	1.903ns	1.255ns
Zeaxanthin	10.089**	6.256*	2.161ns
Lutein	1.372ns	13.164**	0.62ns
Carotene	72.155***	31.022***	68.873***
Caffeic acid	3.263ns	0.443ns	0.019ns
Ferulic acid	13.263**	0.331ns	0.004ns
Protocatechuic acid	8.241*	0.219ns	0.087ns
Catechin	3.142ns	0.811ns	0.247ns
Galic acid	3.242ns	1.823ns	0.097ns
p-Coumaric acid	1.955ns	0.021ns	0.062ns
Resorcinol	3.736ns	0.064ns	0.09ns
B-hydroxy benzoic acid	3.18ns	1.65ns	0.095ns
Chlorogenic acid	3.122ns	0.364ns	0.02ns
Syringic acid	0.567ns	0.026ns	0.092ns
Vanilic acid	0.188ns	0.08ns	0.026ns
Quercetin	14.801**	0.176ns	0.061ns
Quercetrin	8.54*	0.419ns	0.57ns
Luteolin	28.307***	0.333ns	0.096ns
Apigenin	4.355ns	0.081ns	0.042ns
Isoquercetrin	8.7*	0.726ns	0.208ns
Rutin	10.878**	0.619ns	0.168ns

Ellagic acid	46.943***	0.604ns	0.209ns
Velutin	21.377***	0.289ns	0.174ns
Phenols	49.999***	2.768ns	10.189**
Flavonoids	59.598***	1.904ns	0.549ns
PAL	469.272***	14.496**	2.217ns
Antioxidant activity			
FRAP	44.243***	0.883ns	0.233ns
ABTS	10.354**	9.496**	2.358ns
DPPH	24.051***	0.908ns	1.965ns
Anti-Inflammatory activity			
Anti-cyclooxygenase-2	19.513***	46.747***	10.828**
Anti-lipoxygenase	152.183***	6.308*	1.38ns
Hypocholesterolemic activity			
Inhibition of micellar solubility	26.637***	0.146ns	1.806ns
Anti-lipase	1195.932***	94.906***	40.308***
Anti-amylase	185.251***	41.888***	42.81***
Anti-cancer activity			
Hepatocellular carcinoma	23.376***	2.353ns	0.126ns
Colon carcinoma	0.002ns	8.938**	2.79ns
Embryonic kidney adenocarcinoma	51.026***	3.786ns	8.606**
Urinary bladder carcinoma	158.109***	1.26ns	4.351*
Anti-bacterial activity			
Streptococcus sp.	1861.145***	51.015***	13.897***
Escherichia coli	80.593***	19.403***	61.337***
Bacillus subtilis	140.773***	54.668***	35.191***
Pseudomonas aeruginosa	421.357***	3.149**	27.12***
Sarcina lutea	192.555***	17.848***	31.489***

Supplementary Table S2: Levels of antioxidant metabolites, glucosinolates, their amino acid precursors and activities of phenylalanine ammonia lyase (PAL) and myrosinase in the sprouts of three *Lepidium sativum* cultivars grown under either aCO_2 (410 µmol CO_2 mol^{-1} air; control) or eCO_2 (620 µmol CO_2 mol^{-1} air).

Parameters	aCO_2			eCO_2		
	**Haraz**	**Rajab**	**Khider**	**Haraz**	**Rajab**	**Khider**
**Amino acid precursors of glucosinolates and phenolics**						
Alanine	2.75±0.22c	1.58±0.09b	1.12±0.07a	2.19±0.14B*	1.43±0.62A	1.52±0.16A*
Leucine	16.03±1.12a	19.26±1.9b	18.37±0.15ab	24.8±1.23B*	15.72±6.6A*	25.45±1.77B*
Isoleucine	2.7±0.3a	2.58±0.23a	2.45±0.19a	3.14±0.1B*	2.67±0.2A	2.17±0.2A
Valine	8.67±4.4b	8.16±0.13b	3.09±0.18a	21.76±1.2B*	38±4.95C*	6.4±0.41A*
Methionine	13.25±1a	13.8±0.89a	17.3±0.6b	23.87±1.7A*	24.78±1.6A*	21.79±1.4A*
Phenylalanine	6.55±3.3a	25.47±3.3b	7.67±0.05a	14.44±1.15A*	37.38±4.86B*	11.99±1.68A*
Tyrosine	13.77±0.85c	9.95±0.28b	6.68±0.44a	18.37±2.27B*	22.52±1.67B*	14.15±1.37A*
Tryptophane	3.41±0.22a	5.11±0.1b	5.78±0.43b	6.14±0.04A*	10.58±0.62B*	8.36±0.12AB*
**Glucosinolates and its hydrolysis**						
Glucotropaeolin	0.96±0.01ab	0.69±0.16a	1.57±0.07c	1.89±0.18A*	1.72±0.09A*	2.16±0.04A*
Total glucosinolates	46.6±0.9a	47.15±2.0a	69.6±1.2b	88.77±1.49A*	107.9±9.01A*	97.75±6.76A
Benzaldehyde (µg/g)	0.44±0.05a	0.42±0.02a	0.36±0.13a	1.06±0.24A*	1.11±0.23A*	0.84±0.07A*
Benzyl cyanide (µg/g)	0.73±0.01b	0.56±0.01a	1.09±0.02c	1.53±0.1A*	1.35±0.03A*	2.04±0.21B*
Benzyl thiocyanate (µg/g)	1.57±0.1a	1.66±0.09a	2.07±0.17b	4.48±0.3A*	3.48±0.3A*	3.63±0.2A*
Benzyl isothiocyanate [illegible]	3.87±0.08a	3.91±0.17a	5.77±0.1b	7.42±0.08B*	5.15±0.37A*	8.11±0.56B*
**Myrosinase**	3.43±0.31a	4.92±0.25b	9.71±0.38c	7.13±0.36A*	12.56±0.2B*	12.2±0.5B*
**Carotenoids**						
Carotene	0.15±0.012a	0.20±0.00b	0.14±0.00a	0.20±0.01A*	0.26±0.02A*	0.50±0.04B*
Lutein	0.06±0.00a	0.09±0.00a	0.07±0.0a	0.06±0.01A	0.11±0.0B*	0.07±0.01AB
Zeaxanthin	0.001±0.0a	0.001±0.00a	0.002±0.0a	0.002±0.0A*	0.002±0.0A*	0.002±0.0A
Xanthophylls	0.038±0.00a	0.035±0.00a	0.041±0.00a	0.116±0.01B*	0.092±0.0A*	0.102±0.01A [illegible]
**Phenolic compounds**						
Caffeic acid	0.016±0.00a	0.013±0.00a	0.026±0.00b	0.02±0.01A*	0.021±0.01A*	0.01A±0.00*
Ferulic acid	2.00±0.40a	2.33±0.47ab	3.53±0.40c	3.87±0.34B*	3.97±0.39B*	2.35±0.28A*
Protocatechuic acid	1.091±0.21a	1.204±0.17ab	1.729±0.34b	1.98±0.4B*	1.74±0.24B*	1.19±0.11A*
Catechin	0.521±0.1a	0.452±0.12a	0.832±0.16b	0.726±0.20A*	0.526±0.22A*	0.424±0.05A*
Galic acid	13.51±2.7a	18.28±5.36ab	21.62±4.3b	29.19±8.5B*	15.94±6.74AB	10.02±1.2A*
p-Coumaric acid	0.90±0.18a	0.85±0.25a	1.31±0.39b	1.3±0.14A*	1.16±0.19A*	0.92±0.10A*
Resorcinol	0.011±0.0a	0.013±0.0a	0.072±0.05a	0.048±0.02B*	0.066±0.05B*	0.019±0.3A*
p-hydroxy benzoic acid	18.021±3.6a	23.93±7.02ab	28.68±5.92b	38.1±11.2B*	21.38±8.9AB	13.68±5.4A*

Chlorogenic acid	3.97±0.79a	3.22±0.95a	3.152±1.3a	5.1±0.25A*	4.975±0.32A*	6.30±0.12B*
Syringic acid	0.54±0.15a	0.56±0.09a	0.507±0.11a	0.588±0.11A	0.712±0.17B*	0.702±0.10B*
Vanillic acid	0.83±0.24a	0.862±0.3a	0.94±0.32a	0.908±0.3A	1.1±0.52A*	0.903±0.21A
Quercetin	1.196±0.24a	1.075±0.27a	2.94±0.59b	2.563±0.75B*	2.518±0.61B*	1.102±0.18A*
Quercitrin	0.086±0.01a	0.096±0.03a	0.136±0.02b	0.199±0.06B*	0.202±0.05B*	0.066±0.02A*
Luteolin	0.029±0.01a	0.029±0.01a	0.071±0.01b	0.08±0.01AB*	0.085±0.01B*	0.034±0.01A*
Apigenin	0.11±0.023a	0.12±0.024a	0.25±0.087b	0.24±0.01B*	0.207±0.04B*	0.11±0.02A*
Isoquercetin	0.19±0.04a	0.18±0.03a	0.42±0.08b	0.381±0.04C*	0.28±0.02B*	0.149±0.02A*
Rutin	0.83±0.16a	0.78±0.12a	2.18±0.13b	1.96±0.06C*	1.487±0.06B*	0.62±0.26A*
Ellagic acid	0.199±0.03a	0.189±0.03a	0.558±0.08b	0.515±0.07C*	0.457±0.06B*	0.173±0.04A*
Velutin	0.33±0.13a	0.26±0.11a	0.64±0.10b	0.60±0.12B	0.59±0.10B*	0.23±0.07A*
Flavonoids	0.52±0.18a	1.37±0.13b	1.15±0.13b	2.89±0.0A*	3.28±0.05B*	2.87±0.27A*
Total polyphenols	3.33±0.11a	3.66±0.04a	6.67±0.15b	9.65±0.5B*	7.79±0.5A*	14.05±2.7C*
**PAL**	0.91±0.02b	1.10±0.17b	0.79±0.01a	1.97±0.50A*	2.41±0.10A*	2.1±0.10A*

Values are presented as means ± standard error of 5 independent replicates. Different letters in the same row and the same CO_2 level indicate significant difference at the 0.05 probability level as revealed by Tukey's test. Asterisks indicate significant changes ($p < 0.05$) compared to the corresponding control, as revealed by Student’s t-test.

Supplementary Table S3: Antioxidant, anti-inflammatory, hypocholesterolemic, anti-cancer and antibacterial activities of the sprouts of three *Lepidium sativum* cultivars grown under either aCO_2 (410 μmol CO_2 mol^{-1} air; control) or eCO_2 (620 μmol CO_2 mol^{-1} air).

Parameters	aCO_2			eCO_2		
	**Haraz**	**Rajab**	**Khider**	**Haraz**	**Rajab**	**Khider**
**Antioxidant capacity**						
FRAP	6.45±0.7ab	9.25±6.7b	5.09±0.2a	24.74±1.1A*	31.9±3.17A*	28.29±8.5A*
ABTS	0.75±0.05a	1.48±0.02ab	2.05±0.1b	1.84±0.2A*	1.77±0.03A*	2.36±0.1B*
DPPH	7.96±0.5a	10.33±0.0b	9.04±2.1ab	23.24±0.1B*	17.6±0.8A*	15.61±2.8A*
**Anti-inflammatory**						
Anti-cyclooxygenase-2	1.14±0.05a	1.38±0.03b	1.02±0.03a	0.76±0.02B*	1.49±0.1C	0.5±0.03A*
Anti-lipoxygenase	7.39±0.2a	7.69±0.4a	6.49±0.3a	4.8±0.23B*	4.22±0.1AB*	3.8±0.22A*
**Hypocholesterolemic activity**						
Inhibition of micellar	46.7±5.0b	42.9±2.9ab	36.4±8.1a	60.48±3.8A*	59.26±1.9A*	67.08±4.2A*
Anti-lipase	2.95±0.27a	3.1±0.17a	3.79±0.03a	2.07±0.15B*	1.49±0.1A*	2.1±0.29B*
Anti-amylase	1.95±0.1a	1.1±0.2a	1. 9±0.1a	0.97±0.2A*	1.09±0.1A	1.12±0.3A*
**Anti-cancer activity**						
Hepatocellular	56.9±1.4a	53.8±2.9a	50.7±6.9a	73.5±4.7B*	68.3±1.5AB*	63.6±0.7A*
Colon carcinoma	55.1±1.6a	68.3±1.5b	81.5±3.0c	65.5±3.3A*	65.8±3.7A	76.2±4.7B
Embryonic kidney	49.6±0.9a	59.9±2.9b	63.6±2b	74.1±1.7B*	67±2.8A*	72.4±2.8AB*
Urinary bladder	51.4±0.7b	45.4±2.3a	42.8±4.7a	72.1±1.7A*	82.9±4.7B*	76.1±0.4AB*
**Anti-bacterial Activity**						
Streptococcus sp.	11.23±0.36a	13.43±0.59ab	14.19±0.23b	23.83±0.08A*	29.12±0.5C*	26.11±0.17B*
Escherichia coli	21.7±0.23ab	20.38±0.8a	23.67±0.7b	25.18±0.3AB*	32.72±0.9B*	21.97±3.3A
Bacillus subtilis	15.1±0.33a	17.7±0.7b	18.73±0.3b	19.37±0.6A*	30.07±0.9B*	20.82±4.6A
Pseudomonas aeruginosa	16.18±0.4ab	14.51±0.2a	18.33±0.3b	22.78±0.6A*	25.25±0.16B*	22.79±0.5A*
Sarcina lutea	17.09±0.5a	23.4±1.02b	24.72±0.4b	29.95±0.4B*	31.82±0.9B*	26.75±0.4A

Values are presented as means ± standard error of 5 independent replicates. Different letters in the same row and the same CO_2 level indicate significant difference at the 0.05 probability level as revealed by Tukey's test. Asterisks indicate significant changes ($p < 0.05$) compared to the corresponding control, as revealed by Student's t-test.
